# Spatio-temporal patterns of gun violence in Syracuse, New York 2009-2015

**DOI:** 10.1371/journal.pone.0173001

**Published:** 2017-03-20

**Authors:** David A. Larsen, Sandra Lane, Timothy Jennings-Bey, Arnett Haygood-El, Kim Brundage, Robert A. Rubinstein

**Affiliations:** 1 Syracuse University Department of Public Health, Food Studies and Nutrition, Syracuse, New York, United States of America; 2 Syracuse University Department of Anthropology, Syracuse, New York, United States of America; 3 Street Addiction Institute, Syracuse, New York, United States of America; 4 Syracuse City Police Department, Syracuse, New York, United States of America; Stony Brook University, Graduate Program in Public Health, UNITED STATES

## Abstract

Gun violence in the United States of America is a large public health problem that disproportionately affects urban areas. The epidemiology of gun violence reflects various aspects of an infectious disease including spatial and temporal clustering. We examined the spatial and temporal trends of gun violence in Syracuse, New York, a city of 145,000. We used a spatial scan statistic to reveal spatio-temporal clusters of gunshots investigated and corroborated by Syracuse City Police Department for the years 2009–2015. We also examined predictors of areas with increased gun violence using a multi-level zero-inflated Poisson regression with data from the 2010 census. Two space-time clusters of gun violence were revealed in the city. Higher rates of segregation, poverty and the summer months were all associated with increased risk of gun violence. Previous gunshots in the area were associated with a 26.8% increase in the risk of gun violence. Gun violence in Syracuse, NY is both spatially and temporally stable, with some neighborhoods of the city greatly afflicted.

## Introduction

Gunshot violence in the United States of America claims the lives of an estimated 34,000 individuals each year through homicide, suicide, and accidental shooting [1]. The homicide rate in the US has decreased from an estimated 5.5 per 100,000 in 2000 to 4.7 per 100,000 in 2012. This gun homicide rate decline masks potentially fatal gun injuries that do not result in death, due to improvements in trauma care [2]. Such emergency transport and medical care for firearm injuries, however, incurs huge costs, estimated at $17.7 billion per year [3].

Trends in gun violence over the past 30 years saw a large rise in the early 1990s nationally, followed by a decline and eventual steadying off through the 2000’s [4]. These trends vary depending on the location—Chicago had its bloodiest year in nearly two decades in 2016 with nearly 2 murders per day whereas New York City saw a continuation of a dramatic decline in murders [5,6].

Social contagion is the process by which behaviors, attitudes, ideas or beliefs spread through populations. It is easily visualized with fashion, wherein trendsetters promote a new style and that new style begins to spread until it is mainstream. For example men’s hair styles often start in the football leagues of Europe before becoming mainstream in the USA. Social contagion has been used to describe the diffusion of various behaviors including the adoption of novel technology [7], the practice of aggressive behavior [8], and the practice of unethical behavior [9]. Fagan and colleagues first applied the principle of social contagion to gun violence [10], and since gun violence has been compared to an infectious disease [11]. In the social contagion model, the scripts, norms, and beliefs are transmitted through social networks. As nonviolent individuals encounter these scripts, norms, and beliefs in any capacity (as victims, as observers or in another capacity), they may adopt them and perpetuate the violence cycle.

National figures obscure the fact that gun violence is not randomly distributed among the general population. Rather, that violence is heavily clustered in cities among impoverished communities and communities of color [12,13]. Gunshot injury is a principal risk factor for premature death in African American and Hispanic individuals. Among African American’s homicide is the sixth leading cause of death (accounting for 2.8% of all African American deaths). Among Hispanics homicide accounts for 1.7% of deaths, while among white individuals homicide accounts for only 0.4% of deaths [14]. Even within groups at higher risk, gunshot violence is heavily concentrated within small social networks of young men [15,16]. Such clustering may indeed follow the 80/20 rule of thumb for infectious diseases wherein just 20% of the population contributes 80% of the onward transmission of a disease [17]. Beyond clustering within particular populations, gunshot violence clusters in particular locations as small as street corners [18,19].

A recent article found that 62% of the homicides in Chicago can be traced through social networks and implicated as “contagious” homicides [20]. Furthermore, Zeoli et al. found gang-related and revenge homicides clustered across space and time, but robbery-related and intimate-partner violence were randomly distributed across space and time in Newark, New Jersey [21]. Better understanding the spatial patterns and diffusion of gun violence may help in developing more effective policies for preventing said gun violence, particularly when barriers to diffusion are better understood. These articles, however, study gun violence as homicides, rather than gunshot events. Not all gunshots lead to injury, and it is understandable that gun violence without injury may perpetuate more gun violence.

This article describes the epidemiology of gunshot violence (with or without human injury) in the city of Syracuse, New York. It is part of a larger on-going longitudinal research project that is a community-university collaboration addressing trauma due to neighborhood violence. Collaborators include faculty and students of Syracuse University, the Syracuse Police Department, the Street Addictions Institute, Inc., the Trauma Response Team, and Mothers Against Gun Violence [22,23]. (Lane et al. under review)

## Methods

### Study location

The city of Syracuse lies in central New York (43.0469° N, 76.1444° W), and is the fourth largest city in the state of New York. According to the 2010 census the city had a population of 145,170, with an estimated 732,117 people in the greater metropolitan area. The median income for the city of Syracuse is $31,365, with 34.6% of the city’s residents below the federal poverty line. Forty-seven percent of the population is non-White and not Hispanic according to the 2010 census. Syracuse had the highest concentration of poverty among African Americans and Hispanics in the US [24].

### Data

We retrieved incidents of shots fired between the dates of January 2009 and July 2015, inclusive, from the Syracuse City police department crime database. The database contains the following information on each incident of shots fired that has been validated by investigation: date, time, address, type of crime, e.g., whether the shots were related to a burglary or assault, other crime, and the number of people killed. We retrieved census block group information including geographic shape files from the 2010 United States Census. Open Street Maps provided a backdrop for visualization of the gunshots [25].

### Spatio-temporal analysis

We converted the address of each gunshot incident to geo-coordinates using the MMQGIS plug-in for Quantum GIS version 2.0. We then generated 4 random control locations within the city boundaries of Syracuse for each episode of gun violence and conducted the spatial scan statistic of gunshots utilizing a Bernoulli process with month as the time period of analysis. The spatial scan statistic, developed by Martin Kulldorff [26], estimates clustering of phenomena within geographical and temporal bounds. In brief the spatial scan statistic creates a moving window wherein the intensity of the gunshots is calculated inside the window and compared to the intensity of the gunshots calculated outside the window. The window with the greatest differential in the intensity of gunshots is considered the primary cluster. Secondary clusters can also be examined. When including a temporal axis the moving window can be thought of as a cylinder that examines intensity within both space and time within the window compared to outside the window. The spatial scan statistic has been used to examine clusters of violence in Newark, NJ as well as São Paulo, Brazil [11,27]. We used the SatScan software version 9.3 to calculate the spatial scan statistic for gunshots within the city of Syracuse [28]. The use of the Bernoulli process avoided a small numbers problem that would have been introduced by using a Poisson process of gun violence rates of census block groups.

### Factors predicting gunshot violence at the census block-group level

We retrieved census data from the 2010 US census for the city of Syracuse. There were 53 census tracts in Syracuse, divided into 151 census block groups for which demographic data is available. We then conducted a zero-inflated Poisson regression of the number of gunshots per month per census block group with correlated standard errors at the census block group level. A zero-inflated Poisson (ZIP) regression is a two-stage process, first predicting whether there were any gunshots in a census block group or not and then predicting among those with gunshots the number of gunshots in a Poisson distribution. From the available measures in the US census at census block-group level we hypothesized that the following would be associated with gunshots in the census block group: percent of renting households, ratio of empty (vacant) households to occupied households, ratio of female population to male population, and percent of households that identify as either African-American or Hispanic. In the inflation process of the ZIP model we categorized these factors as above or below the median to predict whether there would be any gunshots in a particular census-block-group month. We then incorporated these factors as continuous variables in the Poisson process of the ZIP model. We also controlled for year, whether the month fell between May and October, and the number of gunshots in the census block-group the previous month.

The equations for the ZIP model are presented below wherein y_ij_ is the number of gunshots per census block group (i) during month (j), π is the probability of a census block group having zero shots that month, λ is the Poisson distribution, β represents the covariates included in the analysis as specified above, and h_ij_ is the count of gunshots in a given census block group (i) during month (j).

$$\begin{array}{l} \text{Pr}(y_{ij}=0)=\pi +(1-\pi )e^{-\lambda }e^{\beta }\\ \text{Pr}(y_{ij}=h_{ij})=(1-\pi )\frac{\lambda^{h_{ij}}e^{-\lambda }e^{\beta }}{h_{ij}!} \end{array}$$

We also conducted a sensitivity analysis to determine if the modifiable areal unit problem might be biasing the results from the ZIP model. Gun violence as we have described it herein typically occurs outside rather than in homes, and so aggregating household census data into census block groups better captures neighborhood effects than household census data. To determine if the arbitrary delineation of census block groups affected the results we jittered the location of the each incident of gun violence by a random factor between -462m and 462m on both the x and y axes. We then reran the ZIP analysis and compared the direction, magnitude and significance of results. We used Stata version 13.1 for the regression analysis.

## Results

**Fig 1:**
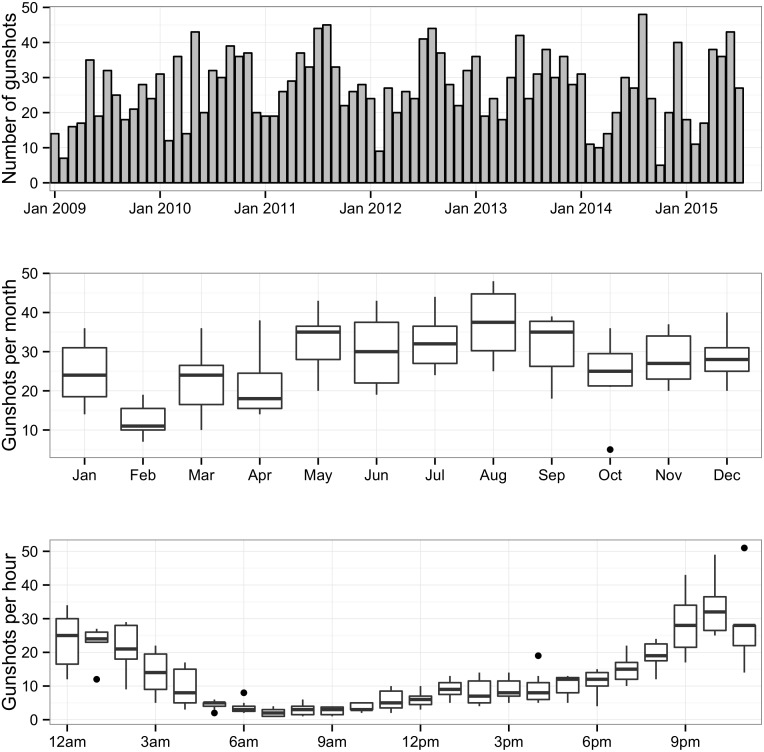
Temporal trends in gunshots in the city of Syracuse, January 2009–June 2015. A) gunshots per month over time, B) gunshots per month each year, C) gunshots per hour each year.

**Fig 2:**
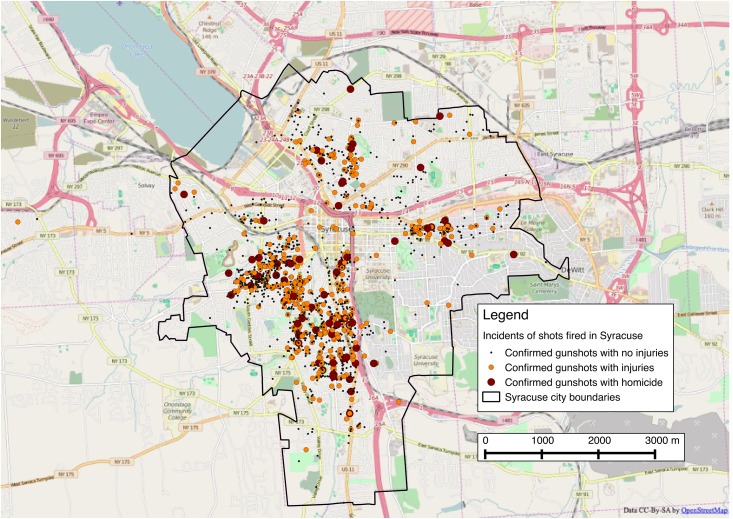
Map of Syracuse, NY depicting gunshots investigated by the police department between January 2009 and July 2015. Map is backdropped against an Open Street Map layer.

**Fig 3:**
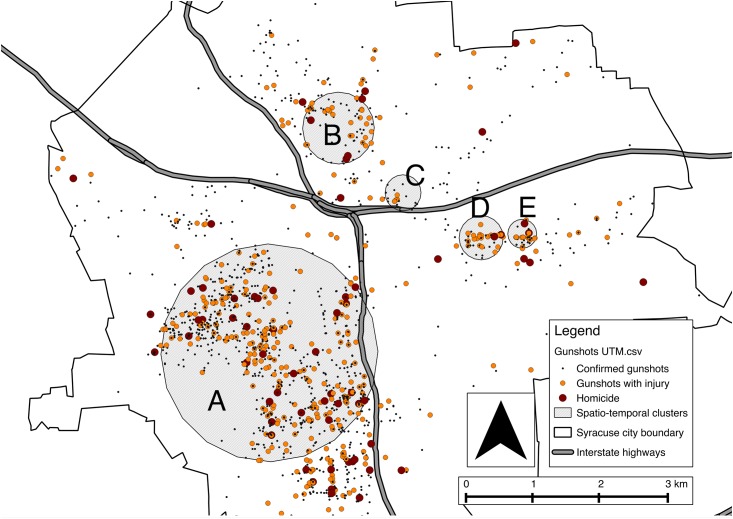
Revealed spatio-temporal clusters of the incidence of gunshots between January 2009 and June 2015. Cluster A was detected from Jan 2009 through July 2012, luster B was detected from Aug 2012 through July 2015, cluster C was detected from Aug 2011 through July 2014, cluster D was detected from January 2009 through July 2012, and cluster E was detected from August 2012 through July 2015.

From January 2009 through June 2015, 2,127 incidents of shots fired within the city of Syracuse were investigated by the Syracuse City Police Department. In 453 of those incidents an individual was injured, and in a further 72 of these incidents an individual was killed. The rate of gunshots per census block group per year ranged from 0 to 16.9. After standardizing per 1,000 population the shots per year range from 0 per 1,000 population to 545 per 1,000 population. Over this time period the number of shots fired within the city each year remained relatively stable with more gunshots in the warmer months May through October and fewer gunshots in the colder months of November through April (Fig 1). Fig 2 shows the location of the gunshots within the city. The spatial scan statistic revealed five statistically significant spatio-temporal clusters (Fig 3).

Census block groups with more rental units, more vacant housing and higher African-American and Hispanic populations had higher numbers of shots fired (Table 1). From the regression analyses these census block groups were at increased risk of having an incident of shots fired (Table 2). Having gunshots within a census block group the previous month also increased the risk of having gunshots within the census block group. Also the warmer months May through October were associated with a significant increase in the risk of gunshots. The sensitivity analysis of jittered gun violence location showed the same patterns in terms of magnitude of effect, direction and significance of each covariate.

**Table 1:** Frequency of gun violence in census block groups January 2009–July 2015.

Factor		Range	Median
Overall		0–92	5
Proportion of houses rented	≤ median	0–85	2
	> median	0–92	13
Proportion of households identifying as AA or Hisp	≤ median	0–28	2
	> median	0–92	22
Ratio of empty to occupied houses	≤ median	0–62	2
	> median	0–92	14
Ratio of females to males	≤ median	0–57	3
	> median	0–92	13

Incidents of gunshots were cumulated over the entire study period.

AA: African American

Hisp: Hispanic

**Table 2:** Results from a zero-inflated Poisson regression model showing factors associated with gunshots in census block group-months.

Factor	Incident rate ratio (95% confidence interval)	P-value
Shots within the census block group the previous month	1.268 (1.181–1.362)	<0.0001
Percentage of houses that are rental units	2.098 (1.089–4.039)	0.027
Ratio of vacant houses to occupied houses	2.379 (0.420–13.486)	0.325
Percent of individuals African-American or Hispanic	11.556 (4.886–27.334)	<0.0001
Months of May–October	1.40 (1.285–1.530)	<0.0001

Inflation factors included rent above or below median, empty houses above or below median, African-American or Hispanic population above or below median, and ratio of female adults to male adults above or below the median. Models also controlled for year.

N = 10,241 census block group-months

## Discussion

Over the course of 5.5 years the city of Syracuse witnessed at least 2,127 incidents of shots fired leading to 72 deaths and a further 453 injuries. Not only is this level of violence quite high, but also some areas experience more than 10 confirmed incidents of gunshots per year. In contrast, 13.5% of census block groups in the city did not experience a single incident of gunshots and 64% of census block groups in the city experienced fewer than two. During this time period, 2009 through 2015, gunshots were stable in space and across time, with increased shooting occurring in the warmer months of the year. It appears that the north side of Syracuse became more violent during the study period.

Each gunshot reported within a census block group the previous month increased the probability of a gunshot being reported by 27%, suggesting that there is a level of endemic violence in certain areas of the city. Due to historical factors leading to segregation in the city these areas are predominantly populated by African-American or Hispanic individuals, and have lower home ownership rates than the rest of the city. The gun violence herein adds to the large burden of health disparities present in these populations, which have been demonstrated in earlier studies by our research team [29]. As has been evidenced elsewhere the higher rate of violence in the African-American and Hispanic census block groups is likely not due to race, but rather due to economic and environmental disparities [30].

During the time period we analyzed in the city of Syracuse there appears to be some diffusion of gun violence from the main centers in the south west quadrant (Fig 3, cluster A) and east side clusters (Fig 3, clusters D, E) to the north (Fig 3, clusters B, C). Indeed earlier Syracuse City Police Department data indicate that the cluster on the eastern side of the city is newer (Fig 3, clusters D, E), coinciding with the recent construction of public housing in that area. The two interstate highways that bisect Syracuse (I-690, I-81) appear to present physical barriers to the diffusion of the gun violence into the south east quadrant of Syracuse. More likely than the interstates themselves stopping gun violence is the reality of the segregation and concentration of poverty in the neighborhoods that are delineated by the interstates. The construction of the interstates during urban renewal was instrumental in disrupting an established African-American community; the existence of the interstates contributes to the continuing devaluation of property within the city of Syracuse. For people living in the surrounding suburbs and towns the interstates present easy access to the hospitals and universities, which are big employers in the city.

Other articles documenting the spatial and temporal trends of violence in urban areas have used either homicides or assaults as the outcome measure. Herein we have used incidents of gunshots to both good and bad effect. Syracuse is a medium-sized city, much smaller than Boston, Chicago, Houston or Newark, for example, where other spatial analyses have been conducted. Although having similar homicide rates as those larger metropolitan areas, the smaller raw numbers of homicides both limit the statistical power of inference and present the small numbers problem when using homicide as the outcome. The use of incidents of gunshots therefore improves the statistical power with which we can make inferences though could still be subject to the small number problem. We used a Bernoulli case-control method to measure spatio-temporal clusters of gun violence rather than the Poisson method to circumvent the issue of small numbers. Further we did not offset the ZIP regression by a population and therefore this analysis was not affected by the small number problem. The ZIP regression is subject to the modifiable areal unit problem, but our sensitivity analysis suggests the results are robust and we do not consider the results limited by these issues.

A certain limitation to using gunshots is that in many cases the occurrence of gunshots is not as definite as the occurrence of homicide or assault where a victim is present. The Syracuse City police department investigated all the incidents of shots fired in the database analyzed herein and discarded those calls which were shown to be something other than gun violence. Furthermore the database of shots fired kept by the Syracuse City police department is not exhaustive of gun violence in the city. Rather it is only the tip of the iceberg as many episodes of gun violence likely go unreported for various reasons. We expect that the spatial and temporal patterns of unreported gun violence are similar to the spatial and temporal patterns of reported gun violence, but in reality there is no way to know.

## Conclusions

The gun violence in Syracuse is heavily concentrated and presents a serious public health problem. Community violence is associated with negative effects for individuals living within the community, even if they are not specifically victims of gunshots. Previous research has found that violence is associated with poor birth outcomes [31], as well as poor cognitive performance [32] and development among children [33]. Our research team is currently developing multi-faceted interventions to respond to this violence [22]. The analyses presented in this article form a foundation for those interventions.
